# The GLP-1 receptor agonist, liraglutide, fails to slow disease progression in SOD1^G93A^ and TDP-43^Q331K^ transgenic mouse models of ALS

**DOI:** 10.1038/s41598-021-96418-0

**Published:** 2021-08-23

**Authors:** Amy Keerie, Heledd Brown-Wright, Isaac Kirkland, Andrew Grierson, James J. P. Alix, Christian Holscher, Richard J. Mead

**Affiliations:** 1 Sheffield Institute for Translational Neuroscience, Department of Neuroscience, School of Medicine, University of Sheffield, Sheffield, UK; 2 Second Hospital, Neurology Department, Shanxi Medical University, Taiyuan, Shanxi People’s Republic of China; 3 Research and Experimental Center, Henan University of Chinese Medicine, Zhengzhou, Henan People’s Republic of China

**Keywords:** Neurochemistry, Peptides, Biochemistry, Neuroscience, Motor control, Neuronal physiology, Drug development, Experimental models of disease, Preclinical research, Translational research

## Abstract

GLP-1 receptor agonists used for the treatment of diabetes, have shown some neuroprotective effects in cellular and animal models of Alzheimer’s disease (AD) and Parkinson’s disease (PD). There are currently few studies investigating GLP-1 receptor agonists in the treatment of ALS, where these neuroprotective effects may be beneficial. Here we investigate the effects of liraglutide, a GLP-1 receptor agonist, in two well characterised transgenic mouse models of ALS (SOD1^G93A^ and TDP-43^Q331K^) to determine if liraglutide could be a potential treatment in ALS patients. Doses of liraglutide previously shown to have efficacy in AD and PD mouse models were used. Behavioural testing was carried out to ascertain the effect of liraglutide on disease progression. Immunohistochemical analysis of tissue was used to determine any neuroprotective effects on the CNS. We found that liraglutide dosed animals showed no significant differences in disease progression when compared to vehicle dosed animals in either the SOD1^G93A^ or TDP-43^Q331K^ mouse models of ALS. We also observed no changes in motor neuron counts or glial activation in lumbar spinal cords of liraglutide treated mice compared to vehicle dosed mice. Overall, we found no evidence to support clinical evaluation of liraglutide as a potential candidate for the treatment of ALS.

## Introduction

There is increasing evidence that Glucagon Like Peptide 1 (GLP-1) Receptor agonists have therapeutic potential in neurodegenerative diseases. These agents were developed for the treatment of diabetes mellitus but several trials are underway in both Parkinson’s disease (PD) and Alzheimer’s disease (AD). These trials were stimulated by extensive preclinical data showing that these agents have neuroprotective and anti-inflammatory properties^1^. However, such data is not available for motor neuron disease. We set out to evaluate this clinically approved, CNS penetrating therapeutic in two mouse models of Amyotrophic lateral sclerosis (ALS). If positive, this data could directly support a clinical trial with liraglutide in ALS.

The concept of exploring the therapeutic utility of anti-diabetic agents in both AD and PD came from multiple independent studies showing that diabetes is a risk factor for these diseases^2,3^. Further evidence at the histological and biochemical level using patient derived material indicated that insulin insensitivity was a general feature of these diseases in the majority of patients, regardless of diabetic status^4–6^. Early studies of insulin resistance in ALS indicated an association of disease with diabetes and later studies found a strong association with type I diabetes but not type II diabetes, indeed some studies have indicated a protective effect of Type II diabetes^7^.

As well as established functions in glucose homeostasis, insulin acts as a growth factor and the receptor is expressed in neurons where it can mediate neurotrophic effects and neuroprotection from stressors including oxidative stress^8^. In terms of therapeutic utility, insulin is problematic as chronic administration in non-diabetics has significant risk. An alternative are anti-diabetic agents such as the GLP-1 mimetics which have been developed to normalise insulin signalling in diabetic patients with great success. Crucially, these molecules have a good safety profile within non-diabetic patients^3^.

It is worth noting that a trial of the anti-diabetic agent pioglitazone (PPARγ agonist) in ALS with the rationale of attenuating inflammation, failed to show any benefit^9^. Importantly this was based on two preclinical efficacy studies in the SOD1^G93A^ mouse which did not account for the now well described pitfalls in experimental design^10^, which have led to new guidelines for preclinical research. In addition, many investigators also advocate testing in additional genetic models, and not just those based on mutant SOD1 overexpression^11^. The use of different model systems increases the confidence in translation from mouse to human studies.

Previous investigations with liraglutide in multiple preclinical AD and PD models have demonstrated neuroprotection. Importantly GLP-1 is brain penetrant^12^, as is liraglutide^13^, which is currently marketed for use in diabetic patients. The GLP-1 receptor is expressed in the CNS in neurons and reactive glia and the effects of liraglutide include inhibition of inflammation and improvement in synaptic connectivity in multiple in vivo models of AD and PD^1^. The predominant effective dose used in mouse studies is 25 nmol/kg. This dose was also shown to reduce the symptoms of Alzheimer’s disease in the APP/PS1 transgenic mouse model^14–16^. Liraglutide treatment in a different mouse model of AD also provided metabolic improvements in the triple transgenic mouse model of AD using ^18^F deoxy glucose-PET studies^17^. These observations are also in keeping with those observed in a recent human clinical trial in AD at the clinically approved doses^18^ and provide an excellent biomarker to match mouse doses to human intervention studies. Liraglutide at this dose furthermore reduced the pathology in the MPTP mouse model of Parkinson’s disease^19^.

Studies in ALS models have also indicated potential beneficial effects of GLP-1 receptor agonism. In vitro studies showed neuroprotective effects of the GLP-1 analog, N-acetyl-GLP-1 (7-34) amide against excitotoxic damage in purified embryonic motor neurons derived from SOD1^G93A^ transgenic mice^20^. In vivo studies have shown a protective effect when mesenchymal stromal cells engineered to produce GLP-1 were transplanted directly into brain ventricles^21^ and the GLP-1 analog exenatide^22^ in an ALS mouse model, however there are still limited studies investigating this in ALS.

In order to gain a better understanding of the role of GLP-1 agonists in ALS and their potential for clinical translation we conducted in vivo pharmacology studies with liraglutide following established guidelines for preclinical testing^11^. We used two strains of transgenic mice which replicate features of ALS, an optimised SOD1^G93A^ transgenic model^23^ and a TDP-43^Q331K^ transgenic line^24^.

## Results

### Liraglutide does not alter behavioural markers of ALS in a TDP-43^Q331K^ model of ALS

**Figure 1:**
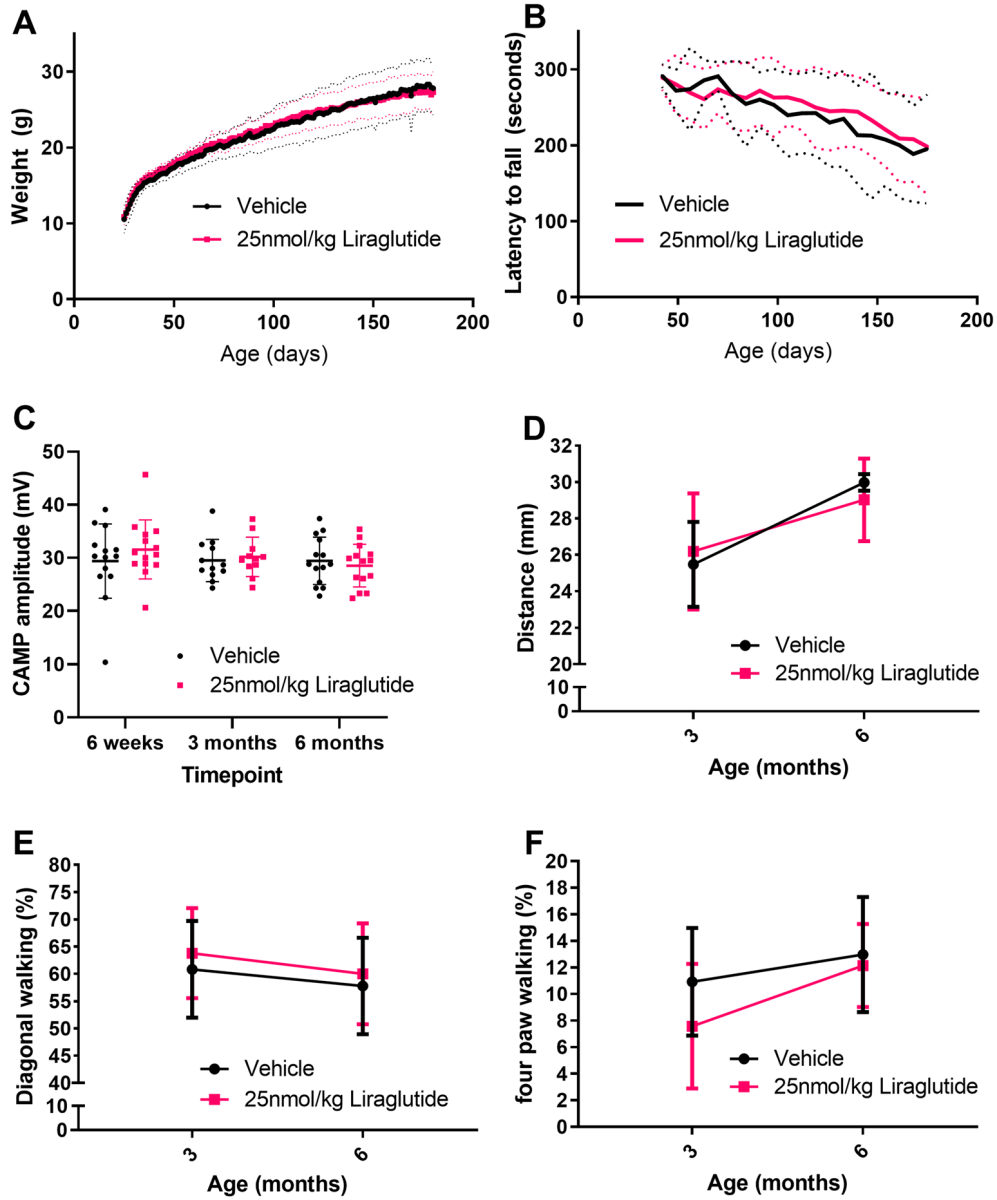
Liraglutide, dosed once daily at 25 nmol/kg IP does not slow disease progression in a TDP-43^Q331K^ transgenic mouse model of ALS. (**A**) Daily average body weight (mean ± SD) of mice dosed with vehicle or liraglutide, once daily, ip from 25 days to 6 months of age. As expected, TDP-43^Q331K^ mice gain weight through to 6 months of age. No significant difference was seen in bodyweight data between the two groups (n = 14 per group, two-way ANOVA with repeated measures *P* = 0.7442). Average shown by thick line, ± SD shown by thin dashed lines (**B**) Rotarod data shown as average latency to fall (mean ± SD) from 50 to 180 days of age. Performance on rotarod declined over time as expected from the model but there was no significant difference between the two dose groups (n = 14 per group, two-way ANOVA with repeated measures *P* = 0.5741). (**C**) Compound muscle action potential (CMAP) amplitude at 6 weeks, 3 months and 6 months of age shown as individual values with mean ± SD. There is no significant difference in CMAP between groups (n = 14 per group, two-way ANOVA with repeated measures *P* = 0.5655). (**D**) Hindlimb base of support (mean ± SD) at 3 and 6 months of age from gait analysis shows no significant difference between dose groups (n = 7 per group, two-way ANOVA *P* = 0.8974 NS). (**E**) Percentage of time spent on diagonal walking (mean ± SD) at 3 and 6 months of age (n = 7 per group, two-way ANOVA *P* = 0.5215 NS). (**F**) Percentage of time spent on four paws (mean ± SD) at 3 and 6 months of age (n = 7 per group, two-way ANOVA *P* = 0.3100 NS).

TDP-43^Q331K^ transgenic female mice were dosed IP daily with vehicle or 25 nmol/kg liraglutide from 25 to 180 days of age. Animals were weighed daily over the duration of dosing. The TDP-43^Q331K^ transgenic mice weigh more than their wild type litter mates and gradually gain weight^25^. All mice in this study put on weight gradually from 25 days of age until the end of the study however, there was no significant difference between the two dose groups. At 30 days of age vehicle and liraglutide animals weighed 13.6 ± 1.4 g and 14.0 ± 0.9 g respectively, and at 6 months vehicle animals weighed 27.9 ± 3.2 g and liraglutide dosed animals weighed 27.0 ± 2.8 g (Fig. 1A) (overall *P* value = 0.7442, two-way ANOVA with repeated measures).

Animals were tested on the rotarod once a week from 6 weeks of age. The TDP-43^Q331K^ transgenic mice dosed with vehicle showed a gradual decline in rotarod performance over the duration of dosing as expected, however there was no significant difference between liraglutide dosed animals and vehicle dosed animals. Vehicle dosed animals on average fell after 291.9 ± 15.1 s at 6 weeks of age and 195.4 ± 71.8 s at 6 months of age and liraglutide dosed animals fell on average after 289.2 ± 16.1 s at 6 weeks of age and 198.1 ± 63.0 s at 6 months of age (Fig. 1B) (overall P value = 0.5741, two-way ANOVA with repeated measures).

Compound muscle action potential (CMAP) is a representation of functional motor axons and muscle fibres. Thus, CMAP amplitude is dependent upon the number of axons, fidelity of neuromuscular transmission, and number/size of muscle fibres. TDP-43^Q331K^ transgenic mice have a lower CMAP than their wild type litter mates at 3 and 6 months of age^25^. The average CMAP for vehicle dosed mice was 29.4 ± 7.0, 29.5 ± 4.0 and 29.5 ± 4.5 at the 6-week, 3-month and 6-month timepoints respectively versus 31.6 ± 5.6, 30.2 ± 3.7 and 28.5 ± 4.0 for liraglutide dosed mice. There was no significant difference between liraglutide and vehicle dose groups at any time point (Fig. 1C) (overall *P* value = 0.5655, two-way ANOVA with repeated measures).

Quantitative gait analysis was carried out at 3 and 6 months of age using the Catwalk gait analysis system (Noldus). Hindlimb base of support (BOS) is the distance between the two hind paws during the step cycle. There is an increase in hindlimb BOS from 3 to 6 months in both groups in the study with no significant difference between the two groups at either time point. Vehicle dosed mice had an average BOS of 25.5 ± 2.3 mm and 30.0 ± 0.6 mm at 3 months and 6 months respectively verses 26.2 ± 3.2 mm and 29.0 ± 2.3 mm for liraglutide dosed animals (Fig. 1D) (overall *P* value = 0.8974, two-way ANOVA with repeated measures). This increase in BOS over time could be due to the increase in weight of these mice or reduced motor coordination.

The general walking pattern of wild-type mice shows a high percentage of time in diagonal walking where opposite forepaw and hindpaw are in contact with the ground at the same time, a higher percentage of time on 3 or 4 paws shows instability. The percentage of time spent on diagonal paws decreases between 3 and 6 months for both vehicle and liraglutide dosed animals but there was no significant difference between the groups. Vehicle dosed mice had an average percentage of time spent on diagonal paws of 60.8 ± 8.6% and 57.8 ± 8.9% at 3 months and 6 months respectively verses 63.8 ± 8.3% and 60.0 ± 9.3% for liraglutide dosed animals (Fig. 1E) (overall *P* value = 0.5215, two-way ANOVA with repeated measures). As well as the decrease in diagonal walking there was an increase in the time spent on four paws, however, there is no significant difference between the two groups. Vehicle dosed mice had an average percentage of time spent on four paws of 10.9 ± 4.0% and 13.0 ± 4.3% at 3 months and 6 months respectively verses 7.6 ± 4.7% and 12.1 ± 3.1% for liraglutide dosed animals (Fig. 1F) (overall *P* value = 0.3100, two-way ANOVA with repeated measures). This decrease in diagonal and increase in 4 paw walking indicates instability and loss of motor coordination as the mice age.

Overall, there was no change in behavioural data between liraglutide dosed and vehicle dosed animals in TDP-43^Q331K^ transgenic mice at a dose level of liraglutide that has shown previous beneficial effects in Alzheimer’s disease and Parkinson’s disease^15,19^. In order to investigate higher dose levels and gain a broader understanding of the potential of liraglutide, a further set of experiments was conducted in SOD1^G93A^ transgenic mice.

### Liraglutide lowers blood glucose levels in SOD1^G93A^ transgenic mice

**Figure 2:**
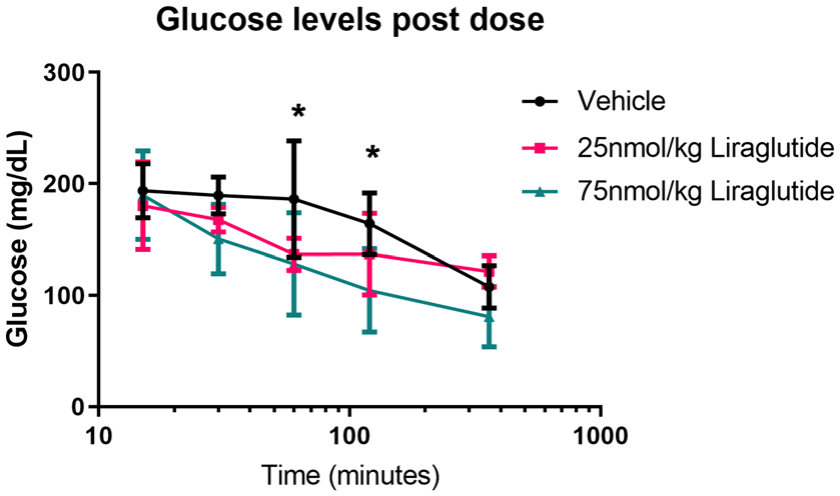
75 nmol/kg liraglutide reduces blood glucose levels in SOD1^G93A^ mice. Blood glucose levels measured on an optium geo glucose meter at multiple time points post dose with vehicle, 25 and 75 nmol/kg liraglutide (mean ± SD). There is a significant decrease in glucose levels at 60 (*P* = 0.0285) and 120 (*P* = 0.0236) minutes post dose in the 75 nmol/kg liraglutide group compared to vehicle (n = 4 per group, two-way ANOVA).

Following on from the TDP-43^Q331K^ mouse study a target engagement study was carried out in SOD1^G93A^ transgenic mice. This was to determine if the dose of liraglutide was affecting glucose levels at the previously used 25 nmol/kg liraglutide dose and a higher dose of 75 nmol/kg liraglutide. Mice were dosed with vehicle, 25 and 75 nmol/kg liraglutide IP and a small amount of blood was collected from the tail at multiple time points to determine blood glucose levels. Baseline glucose levels increased at 15 min post dose for all groups, probably due to stress of handling and dosing. Over the next 24 h the glucose levels decreased in all of the dose groups in a dose dependant manner with the vehicle group having the slowest decline in glucose level over time. There was a significant decrease in blood glucose levels of the 75 nmol/kg liraglutide dosed group when compared to the vehicle dosed group at 60 (*P* = 0.0285) and 120 (*P* = 0.0236) minutes post dose (Fig. 2) (two-way ANOVA with repeated measures).

### Liraglutide does not alter behavioural markers of ALS in SOD1^G93A^ transgenic mice

**Figure 3:**
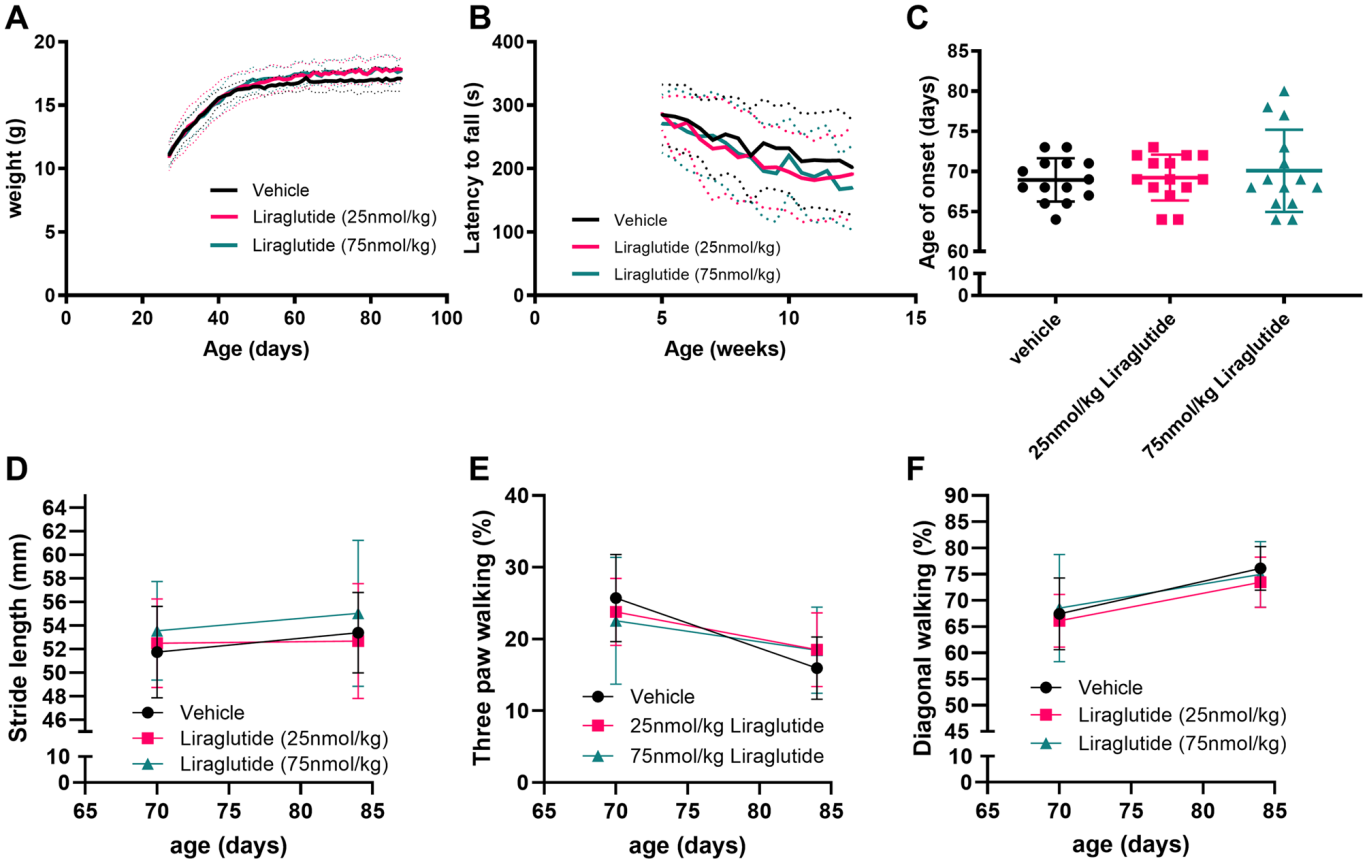
Liraglutide dosed once daily at 25 or 75 nmol/kg does not alter progression of disease in a SOD1^G93A^ mouse model of ALS. (**A**) Daily average body weight (mean ± SD) from 25 to 90 days of age of mice dosed with vehicle, 25 or 75 nmol/kg liraglutide once daily ip. Average shown as thick line and SD shown as thin dashed lines. No significant difference in body weight between liraglutide dosed and vehicle dosed animals (n = 14 per group, two-way ANOVA, *P* = 0.3165). (**B**) Rotarod data shown as latency to fall (mean ± SD) from 40 to 90 days of age. As expected, rotarod performance declined over time but there was no significant difference between vehicle and liraglutide dosed mice (n = 14 per group, two-way ANOVA, *P* = 0.5252). (**C**) Age of visible onset measured through twice weekly motor scoring shown as individual values with mean ± SD (n = 14 per group, one-way ANOVA, *P* = 0.7026). (**D**) Hindlimb stride length (mean ± SD) at 70 and 84 days of age showed no difference between liraglutide dosed and vehicle dosed animals (n = 8 per group, two-way ANOVA, *P* = 0.1298). (**E**) Percentage of time spent on diagonal paws (mean ± SD) at 70 and 84 days of age showed no difference between vehicle dosed and liraglutide dosed mice (n = 8 per group, two-way ANOVA, *P* = 0.7074). (**F**) Percentage of time spent on three paws (mean ± SD) at 70 and 84 days of age (n = 8 per group, two-way ANOVA, *P* = 0.9653).

Due to the tolerated higher 75 nmol/kg liraglutide dose and the larger effect on blood glucose levels, we dosed SOD1^G93A^ transgenic mice with vehicle, 25 nmol/kg or 75 nmol/kg liraglutide from 25 to 90 days of age. From 25 to 45 days of age mice rapidly put on weight, which then plateaued for the duration of the study (Fig. 3A). There was no overall difference between the liraglutide dosed animals and vehicle dosed animals in terms of weight. At 88 days of age vehicle, 25 nmol/kg and 75 nmol/kg dosed animals weighted 17.1 ± 0.9 g, 17.8 ± 1.1 g and 17.6 ± 1.2 g respectively (overall *P* = 0.3165, two-way ANOVA with repeated measures).

Rotarod testing was carried out twice a week from 6 weeks of age. There was a steady decline in rotarod performance from the 6-week time point, as expected in the model; however, there was no significant difference between the different groups when compared to vehicle dosed animals. The average time to fall for vehicle dosed mice was 284.8 ± 47.6 s and 212.9 ± 80.6 s at 5 weeks and 12 weeks respectively, verses 286.4 ± 25.9 s and 187.1 ± 62.9 s for 25 nmol/kg liraglutide dosed mice, and 270 ± 45.7 s and 167.5 ± 55.3 s for 75 nmol/kg liraglutide dosed mice (Fig. 3B, overall *P* = 0.5252, two-way ANOVA with repeated measures).

Onset of visible signs was determined from the combined onset of hind limb splay defects and tremor. There was no significant difference in age of disease onset between the liraglutide dosed animals and vehicle dosed animals. Average age of visible signs of onset for vehicle, 25 nmol/kg liraglutide dose animals and 75 nmol/kg liraglutide dosed animals was 68.9 ± 2.7 days, 69.2 ± 2.9 days and 70.1 ± 5.1 days respectively (Fig. 3C, overall *P* = 0.7026, one-way ANOVA with Sidak’s multiple comparison).

Quantitative gait analysis was carried out at 70 and 84 days of age. No significant difference was observed between liraglutide dosed and vehicle dosed mice in hindlimb stride length (*P* = 0.1298, Fig. 3D), percentage of time spent on diagonal paws (*P* = 0.7074, Fig. 3E) or percentage of time spend on three paws (*P* = 0.9653, Fig. 3F) (two-way ANOVA with repeated measures).

### Liraglutide shows no effect on motor neuron count, or glial responses in the lumbar spinal cord of SOD1^G93A^ transgenic mice

**Figure 4:**
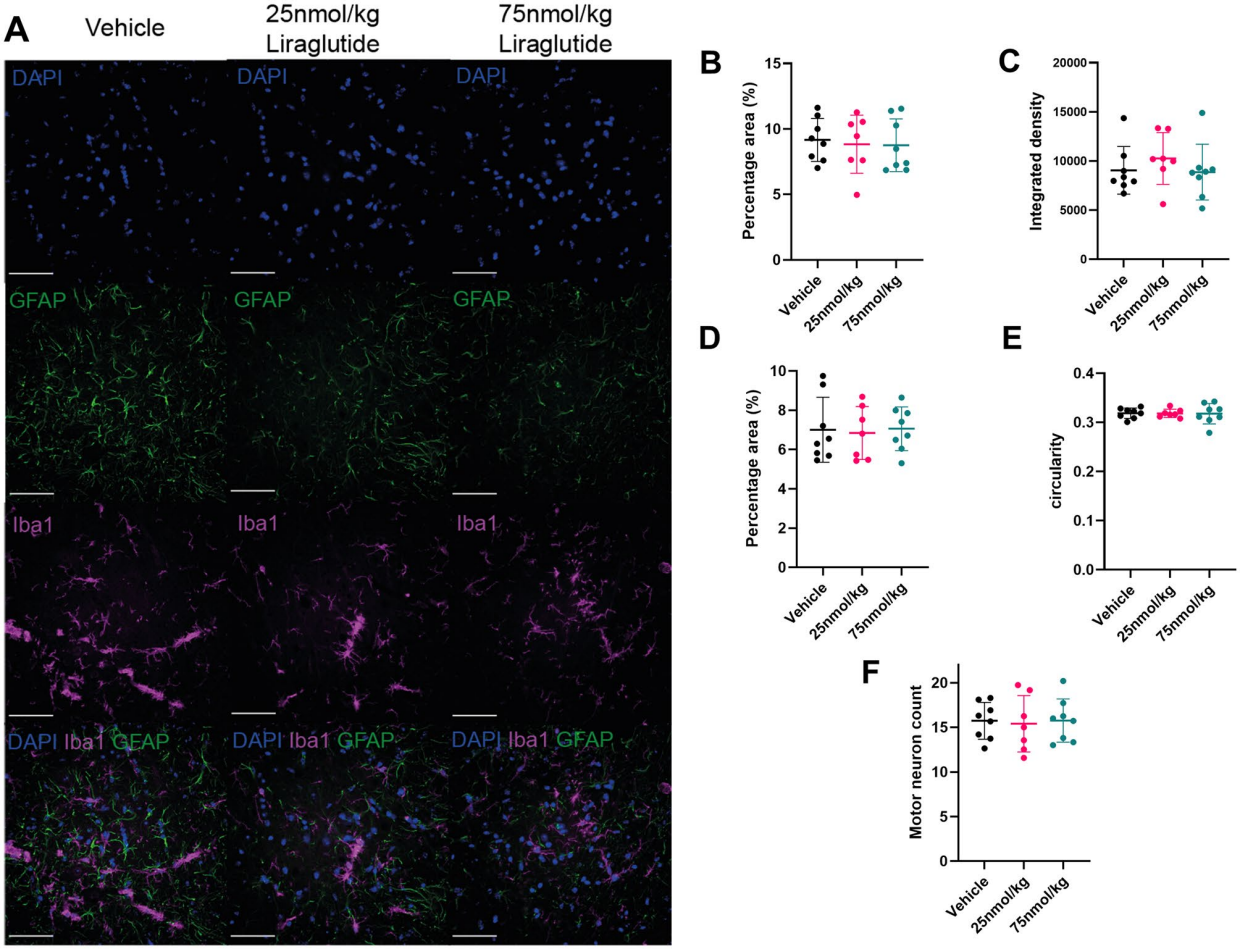
GFAP, Iba1 and Nissl staining of SOD1^G93A^ lumbar spinal cord shows no difference between liraglutide and vehicle dosed animals in a SOD1^G93A^ mouse model. All fluorescence images were captured on the INCELL 2000 (GE) at 60 × magnification. (**A**) Representative images of DAPI, Iba1 and GFAP staining of ventral horns from lumbar spinal cord sections from vehicle, 25 nmol/kg and 75 nmol/kg liraglutide dosed SOD1^G93A^ mice at 90 days of age. Scale bar is 50 μm. (**B**) Quantification of percentage area coverage of GFAP staining plotted as individual values with mean ± SD shows no significant difference between vehicle and liraglutide dosed mice (n = 7 or 8 per group, one-way ANOVA, *P* = 0.7824). (**C**) Integrated density of GFAP staining plotted as individual values with mean ± SD shows no significant difference between liraglutide and vehicle dosed groups (n = 7 or 8 per group, one-way ANOVA, *P* = 0.9754). (**D**) Iba1 percentage area coverage plotted as mean ± SD shows no significant difference between liraglutide and vehicle dosed mice (n = 7 or 8 per group, one-way ANOVA, *P* = 0.7107) (**E**) Circularity of Iba1 staining shown as individual values with mean ± SD shows no significant difference between liraglutide and vehicle dosed animals (n = 7 or 9 per group, one-way ANOVA, *P* = 0.9927). (**F**) Motor neuron counts from Nissl stained lumbar spinal cord sections shown as individual values with mean ± SD shows no significant difference between liraglutide and vehicle dosed animals (n = 7 or 8 per group, one-way ANOVA, *P* = 0.9582).

Since there was no effect of liraglutide on behavioural parameters we examined pathology in lumbar spinal cord from the end of the study when the SOD1^G93A^ transgenic mice were 90 days old. We first looked at astrocyte and microglia staining of lumbar spinal cord sections using GFAP and Iba1 antibodies respectively (Fig. 4A). There was no significant difference in GFAP percentage area stained with 9.2 ± 1.6%, 8.8 ± 2.2% and 8.8 ± 2.0% area coverage for vehicle dosed, 25 nmol/kg and 75 nmol/kg liraglutide dosed respectively (*P* = 0.7824, one-way ANOVA) (Fig. 4B). There was also no significant difference in integrated density of GFAP staining between vehicle and 25 nmol/kg or 75 nmol/kg liraglutide with values of 9047 ± 2425, 10,258 ± 2630 and 8866 ± 2949 respectively (*P* = 0.9754, one-way ANOVA) (Fig. 4C).

There was also no significant difference in area stained for Iba1 with 7.0 ± 1.7%, 6.8 ± 1.3% and 7.1 ± 1.1% area coverage for vehicle, 25 nmol/kg and 75 nmol/kg liraglutide dosed animals (*P* = 0.7107, one-way ANOVA) (Fig. 4D). Nor was there a difference in circularity of Iba1 staining with values of 0.32 ± 0.01, 0.32 ± 0.01 and 0.32 ± 0.02 for vehicle, 25 nmol/kg and 75 nmol/kg liraglutide respectively (*P* = 0.9927, one-way ANOVA) (Fig. 4E).

Motor neurons counts from lumbar spinal cord sections stained with Nissl at 90 days of age showed no significant difference in the average number of motor neurons per ventral horn between the two liraglutide dosed groups and the vehicle dosed group. The vehicle, 25 nmol/kg, and 75 nmol/kg dosed mice had an average of 15.7 ± 2.1, 15.4 ± 3.2 and 15.8 ± 2.4 motor neurons per section respectively (*P* = 0.9582, one-way ANOVA) (Fig. 4F).

## Discussion

We investigated the effect of liraglutide, a GLP-1 receptor agonist, in two mouse models which recapitulate some features of ALS pathophysiology and found no evidence that disease progression was altered at the behavioural or pathological level.

The SOD1^G93A^ mouse model is the most commonly used mouse model to test potential therapeutics for ALS however, it has poor translation to human studies^26^ and may only represent patients with SOD1 mutations. The TDP-43^Q331K^ mouse model used in this study may be more representative of ALS patients and also shows signs of having an FTD-like phenotype with remarkable similarities to a more physiological knock-in model^25^. However, this model does not have the TDP-43 proteinopathy found in the majority of ALS patients^24^. The use of two different mouse models and adherence to the published preclinical guidelines for ALS^11^ gives more confidence in the lack of effect for liraglutide.

GLP-1 receptor agonists increase insulin levels and therefore lower blood glucose levels and insulin plays an important role in CNS mediated regulation of glucose and energy homeostasis as well as on memory, and mood^27^. However, liraglutide may also have an effect on several other pathways linked to neuroprotection^28^. Although GLP-1 receptor agonists have shown a range of neuroprotective properties, there is a clearer link between insulin pathway deficiencies in AD and PD compared to ALS. For example, obesity is a risk factor for AD and disruption of insulin signalling in the brain leads to a higher risk of AD^29^, however ALS patients tend to be hypermetabolic and it has been shown that increasing glucose levels in models of ALS is protective^30^. There is less evidence connecting insulin signalling and risk of ALS as there is with AD and PD.

A previous study looked at a different GLP-1 receptor agonist, exendin-4, in a SOD1^G93A^ mouse model and found no change in disease progression as measured by rotarod performance and a decrease in running wheel but a greater than threefold increase in motor neuron count both pre-onset and at end-stage^22^. These results are difficult to reconcile since the motor phenotype in SOD1^G93A^ mice is clearly linked to motor neuron degeneration and loss. We saw no change in disease progression and no such preservation of motor neurons upon liraglutide treatment. Both the Li et al. study and the present study investigated astrocyte reactivity by GFAP staining, and we did not observe the decrease of GFAP staining seen in the Li et al. study. A second study investigated delivery of GLP-1 in a SOD1^G93A^ mouse model using an intracerebroventricular injection of mesenchymal stem cells (MSC) stably transfected to secrete GLP-1^21^. A significant improvement in weight, survival and rotarod performance was seen in the MSC/GLP-1 treated animals compared to vehicle treated animals. Although this suggests neuroprotective effects of GLP-1, it is not possible in this study to separate protective effects of the mesenchymal stem cells themselves, from the effect of liraglutide, since a non-GLP transfected MSC control was not used. Indeed, we and others have shown direct protective effects of mesenchymal (bone marrow or adipose derived) stem cells in mutant SOD1 transgenic mouse models of ALS^31,32^.

We cannot exclude the possibility that liraglutide may show beneficial effects on, for example, synaptic connectivity, as described in other models^33,34^. However, the data presented here in conjunction with clinical data on this mechanism in ALS suggests that it may not be of value to perform clinical studies with GLP-1 agonists in ALS and that resources may be more usefully focussed elsewhere.

## Materials and methods

### Ethics statement

All animal studies were carried out in accordance of the UK Animals (Scientific Procedures) Act 1986 under a UK Home Office project license. This license was ethically reviewed and approved by the local ethics committee (University of Sheffield Animal Welfare and Ethical Review Body). Mouse maintenance and day to day care was in line with the Home Office Code of Practice for Housing and Care of Animals Used in Scientific Procedures. The ARRIVE guidelines^35^ were followed in the preparation of this paper.

### Transgenic mice

The SOD1^G93A^ C57BL/6 transgenic mice were as described in a previous paper^23^. In brief B6SJL-Tg(SOD1-G93A)1Gur/J mice were backcrossed for at least 20 generations onto the C57BL/6 J OlaHsD background.

TDP-43^Q331K^ C57BL/6 N transgenic mice were obtained from the Jackson Laboratory, stock number 01793 and were originally generated in the Cleveland lab^24^ and extensively characterised in-house^25^.

Mice were housed in groups of between 2 and 5 with ad libitum access to food (standard rodent diet 2018, Evigo) and water. The temperature was maintained at 21 °C with a 12-h light/dark cycle. One plastic house was provided per cage, sawdust (Datesand) was used to cover the floor of the cages and paper wool (Datesand) was provided for nesting material.

### Genotyping

Mice were ear clipped for identification and genotyping. DNA was extracted from earclips using 20 μl of Quickextract (Lucigen) and incubating at 65 °C for 15 min followed by 2 min at 98 °C.

Genotyping of TDP-43^Q331K^ transgenic mice was carried out as described by Jackson Laboratory^36^. Genotyping of SOD1^G93A^ transgenic mice was carried out as previously described^23^.

### Study design

For all the studies female transgenic mice were block randomised into the different dose groups. Power analysis (G Power version 3.0.3) was carried out on both models in order to determine the number of mice required to see a 20% improvement in rotarod performance. For the TDP-43^Q331K^ and SOD1^G93A^ transgenic mouse studies 14 mice were used per dose group.

At the end of the study mice were euthanised with 2.5 ml/kg pentobarbitone. Under deep anaesthesia half of the animals were then perfused with ~ 5 ml PBS followed by 4% PFA, brain and spinal cord were collected for IHC analysis. The other half were euthanised with 2.5 ml/kg pentobarbitone followed by collection of blood, brain and spinal cord that were snap frozen.

### Glucose measurements

Mice were not fasted before the start of the glucose experiment and had access to food ad libitum. A small amount of blood (6 μl) was collected directly onto a test strip from the tail of mice and analysed using an Optium Neo glucose testing kit (FreeStyle).

### Liraglutide and dosing IP

Liraglutide was dissolved in sterile PBS at 0.037 mg/ml, aliquoted and frozen at -20 °C. On the day of dosing an aliquot was defrosted and diluted in PBS to 2.5 and 7.5 nmol/ml. Mice were dosed via intraperitoneal injection at 10 ml/kg. No adverse reactions were detected when dosing at 25 nmol/kg or 75 nmol/kg.

### Rotarod

Rotarod testing was carried out using the protocol described previously^23^. TDP-43^Q331K^ transgenic mice were tested once a week and SOD1^G93A^ transgenic mice were tested twice a week from 6 weeks of age.

### Catwalk gait analysis

Gait was captured using the catwalk gait analysis system 7.1 (Noldus). Mice voluntarily ran across a glass platform, and a camera mounted below captured the footsteps. Up to six runs were recorded for each mouse at each timepoint and the three straightest, most consistent runs are selected for analysis. Image analysis of the gait pattern was carried out using the Catwalk analysis software (Noldus). The gait parameters analysed for each model correlated with those previously identified as being informative for tracking with disease progression^23,25^.

### Neurological deficit scoring

SOD1^G93A^ animals were scored three times a week for neurological deficit from 60 days of age as previously described^23^. Onset of visible signs of disease is defined as a splay defect combined with an abnormal gait score for two consecutive scoring days.

### Electrophysiology

Electrophysiology was carried out at 6 weeks, 3 months and 6 months of age in the TDP-43^Q331K^ transgenic mice. Mice were anesthetised using 5% gaseous isoflurane and 4 L/min oxygen flow rate. Once anesthetised animals were moved to a nose cone maintained at a 2% gaseous isoflurane and 0.5 L/min oxygen flow rate. The left hind limbs were shaved and hair removal cream was used in order to remove all the fine fur. Ring electrodes were placed around the hind limb using Ten20 nerve conductive paste to ensure good contact with the skin. A ground electrode (Ambu Neuroline) was placed in the base of the tail and a pair of stimulating electrodes (Ambu Neuroline) were placed on the skin above the sciatic nerve at the level of the sciatic notch. Recordings were made using a Dantec Keypoint Focus EMG system. A square pulse of 0.1 ms duration was used and the current gradually increased to ensure a supramaximal compound muscle action potential (CMAP) response. The amplitude of the compound muscle action potential (CMAP) was used and is a measurement of the output of all the stimulated axons and their target muscle.

### Preparation of lumbar spinal cord sections

Mice were perfused with 4% PFA in PBS, spinal cord dissected and post-fixed for 24 h. PFA fixed lumbar spinal cords (L3-L4) were cut in half and embedded in paraffin. Sections were cut at 10 μm thickness and placed onto glass slides. Immunohistochemistry (IHC) was carried out on 8 serial sections, 50 μm apart while Nissl staining was performed on a total of 16 serial sections, 50 μm apart. Sections were de-parrafinised and rehydrated by washing slides for 5 min in the following: 2 × 100% xylene, 100% ethanol, 95% ethanol, 70% ethanol and H_2_O.

### Nissl staining

Rehydrated sections were incubated in a 0.1% cresyl violet solution for 15 min. Slides were then rinsed in H_2_O and incubated in acetic acid for 4–8 s. Slides were then dehydrated in 100% ethanol for 1 min and cleared in 100% xylene prior mounting with DPX mountant. Images were collected using a nanozoomer digital slide scanner (Hamamatsu) and analysed in NDP.view 2 software (Hamamatsu). Motor neurons were then counted using a criteria of > 20 μm in cell body size in any axis, location I the ventral horn and presence of a nucleus and nucleolus. Counting of motor neurons was conducted blinded.

### Iba1 and GFAP staining

Sections were deparrafinised and rehydrated as above and subjected to antigen retrieval at pH 9 using Access super RTU (Menarini) for 30 min at 125 °C with 20 psi. After antigen retrieval, sections were cooled and washed in PBS for 5 min. Sections were then blocked and permeabilised using 5% BSA (Sigma Aldrich) and 0.25% triton 100 (Thermo Scientific) for 20 min. Then incubated with the primary antibodies 1 in 500 dilution in 1% BSA and 0.25% triton 100 overnight at 4 °C. Primary antibodies were anti-GFAP raised in chicken (abcam, ab4674) and anti-Iba1 raised in rabbit (GeneTex, GTX100042).

Primary antibodies were washed off with 6 quick PBS washes and 3 washes in PBS with agitation. The slides were then incubated with the secondary antibodies 1 in 1000 for 1.5 h at room temperature. Secondary antibodies were alexa flour anti-rabbit 555 raised in donkey (Thermo Fisher, A27039) and Alexa fluor anti-chicken 488 raised in donkey (Thermo Fisher, A11039). Secondary antibodies were removed with 2 quick PBS washes and 4–8 min washes in PBS with agitation. Slides were the mounted using hard set vectashield with DAPI (Vector Laboratories) and cover slips were added. Fluorescent images were captured using the INCELL 2000 (GE) at a magnification of 60x. Images were analysed using ImageJ where the background was subtracted using a rolling ball and a default threshold (“Li”) was used to detect both GFAP and Iba1 staining. This threshold was then used as a mask to analyse the original image. This method was adapted from Healy et al.^37^.

## Data Availability

The datasets generated during and/or analysed during the current study are available from the corresponding author on reasonable request.
